# Supplementary material for: MOFs/Ketjen Black-Coated Filter Paper for Spontaneous Electricity Generation from Water Evaporation
Source: Polymers (Basel). 2022 Aug 26;14(17):3509. doi: 10.3390/polym14173509 (PMC9459984; doi:10.3390/polym14173509)
Supplement: Supplementary file 1 [file polymers-14-03509-s001.zip › polymers-1883621-supplementary.pdf]

Supplementary Materials for

MOFs/Ketjen Black-Coated Filter Paper for Spontaneous Electricity Generation from Water Evaporation

Jingyu Li, Yexin Dai, Shipu Jiao and Xianhua Liu *

School of Environmental Science and Engineering, Tianjin University, Tianjin 300354, China
* Correspondence: lxh@tju.edu.cn; Tel.: +86-22-85356239

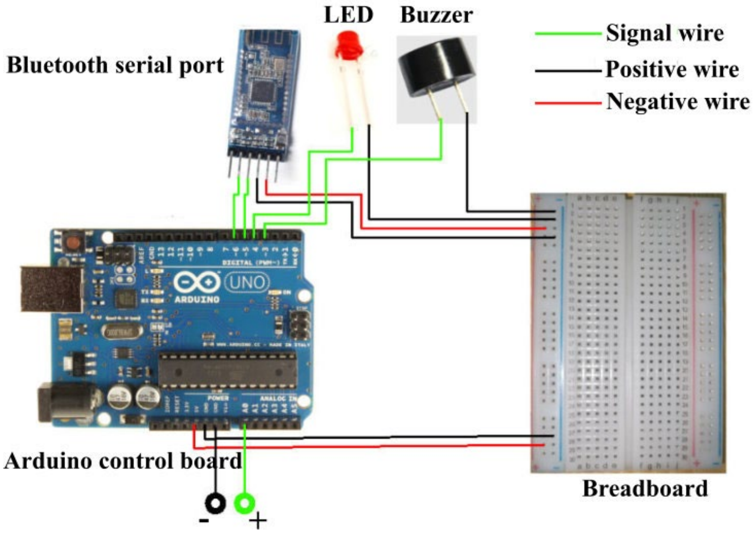

Figure S1. The connection diagram of Control system.

Calculation of Maximum Output Power

For a device with a size of 5 cm (L) × 1 cm (W), the V_{oc} and I_{st} can be found in Figure 4a in the text, and they are ~ 0.28 V and ~ 22.5 μA, respectively.

$$P_{max} = 1/4 \times V_{oc} \times I_{st} = 1/4 \times 0.285V \times 22.5\mu A \approx 1.603\mu W \quad (S1)$$
